# Microbially Synthesized Polymer-Metal Nanoparticles Composites as Promising Wound Dressings to Overcome Methicillin-Resistance *Staphylococcus aureus* Infections

**DOI:** 10.3390/polym15040920

**Published:** 2023-02-12

**Authors:** Jennifer Balcucho, Diana M. Narváez, Natalia A. Tarazona, Jinneth Lorena Castro-Mayorga

**Affiliations:** 1 Nanotechnology and Applied Microbiology Research Group (NANOBIOT), Department of Biological Sciences, University of the Andes, Bogota 111711, Colombia; 2 Human Genetics Laboratory, Department of Biological Sciences, University of the Andes, Bogota 111711, Colombia; 3 Institute of Active Polymers, Helmholtz-Zentrum Hereon, Kantstraße 55, 14513 Teltow, Germany; 4 Agricultural Bioproduct and Bioprocess Research Group, Department of Bioproduct, Colombian Corporation of Agricultural Research, Agrosavia, Mosquera 250047, Colombia

**Keywords:** polyhydroxyoctanoate, antimicrobial films, silver nanoparticles, methicillin-resistant *Staphylococcus aureus*, antimicrobial activity, wound healing

## Abstract

Antimicrobial resistance has been declared one of the top 10 global public health threats. Methicillin-resistant *Staphylococcus aureus* (MRSA) is a leading cause of recurring skin and soft tissue infections in patients with chronic skin conditions such as diabetic foot infections, making the treatment of the ulcers challenging. Wound dressings combined with metal nanoparticles have been suggested to prevent and treat MRSA-infected wounds. However, these particles are commonly synthesized by chemical approaches. In this study, we developed bio-based silver (Bio-AgNPs) and copper oxide nanoparticles (CuONPs) polymer composites using a microbially produced polyester from the Polyhydroxyalkanoates (PHAs) family. Poly(3-hydroxyoctanoate)-*co*-(3-hydroxyhexanoate) (PHO) was synthesized by *Pseudomonas putida* and functionalized in-situ with Bio-AgNPs or ex-situ with CuONPs. PHO-CuONPs films did not inhibit MRSA growth, while a reduction of 6.0 log CFU/mL was achieved with PHO-Bio-AgNPs synthesized from silver nitrate (AgNO_3_) solution at 3.5 mM. Exposure of human fibroblast cells (HFF-1) to the bioactive films did not induce notable cytotoxicity and genotoxicity, as seen by a viability higher than 79% and no significant changes in basal DNA damage. However, exposure to PHO-Bio-AgNPs induced oxidative DNA damage in HFF-1 cells. No hemolytic potential was observed, while platelet aggregation was promoted and desired for wound healing. Here we demonstrate the biosynthesis of polymer-nanoparticle composites and their potential as bioactive films for MRSA treatment.

## 1. Introduction

*Staphylococcus aureus* is a major colonizer of skin lesions, both in the community and in healthcare facilities. Methicillin-resistant *Staphylococcus aureus* (MRSA) has been recognized as one of the most virulent strains causing recurring skin and soft tissue infections (SSTIs) in patients with chronic skin conditions such as atopic dermatitis [1], diabetic foot infections (DFIs) [2], or persons with invasive medical devices or compromised immune systems [3]. MRSA is endemic in many healthcare facilities throughout the world, and consequently, it has become a major focus for infection control efforts globally. In several cases, *S. aureus* (including MRSA) infections become a burden of disease requiring long-term treatment due to complications such as abscesses and osteoarticular infections, among others. For patients with DFI, skin ulcers colonized by MRSA can cause delays in healing and the development of chronic conditions that result in leg amputations [4]. Management of MRSA varies depending on geographical regions, local MRSA prevalence, and the availability of newer antimicrobials effective against MRSA (delafloxacin and omdacycline were recently approved for SSTIs [3]. However, MRSA has demonstrated remarkable resistance to antibiotics over the years, and successful treatment remains challenging. Current strategies for MRSA treatment include the evaluation of both novel antimicrobials and adjunctive aspects of care, such as infectious disease consultation, echocardiography, and source control [5].

Silver nanoparticles and other metals like zinc and copper have been suggested as potent antimicrobial agents and have been immobilized in polymeric matrices used in biomedicine to treat infected wounds [6,7,8]. However, the antimicrobial mechanism of silver is not yet fully understood, with the three most common hypotheses proposed being: (1) uptake of free silver ions followed by disruption of adenosine triphosphate (ATP) production and deoxyribonucleic acid (DNA) replication; (2) silver nanoparticle and silver ion generation of ROS; and (3) silver nanoparticle direct damage to cell membranes [9,10].

Metal nanoparticle-based antimicrobial agents are commonly synthesized by a chemical approach in which reducing agents of organic and inorganic nature are used, such as sodium citrate and *N,N*-dimethylformamide (DMF), the latter of which is a substance of high concern for human health listed by the European Chemicals Agency [11]. Therefore, greener procedures for the synthesis of metallic nanoparticles have been developed, in which natural compounds such as plant extracts, ascorbic acid, and microbial biomass are used as reducing agents [12,13,14]. Several studies have shown that a wide variety of bacteria possess the ability to use biomass as a reducing agent, in particular species of the genera *Lactobacillus* and *Bacillus*, some strains of *Escherichia coli,* and *Alcaligenes eutrophus*, previously known as *Cupriavidus necator* [15,16]. Biologically synthesized silver nanoparticles (Bio-AgNPs) have been successfully incorporated into synthetic polymers commonly used in biomedicine, such as polycaprolactone (PCL) [8], and more recently into bio-based polymers from the family of polyhydroxyalkanoates (PHAs) [16,17,18,19,20], opening a new field of synthesis of antimicrobial wound dressings.

PHAs are a family of biodegradable biopolymers with minimal tissue toxicity that are produced by numerous bacteria as intracellular carbon and energy reserves [21,22]. When comparing these bio-based plastics to some well-studied petroleum-based plastics that can be degraded by microorganisms, such as PCL and polylactic acid (PLA), PHAs presented several advantages since their physical and mechanical properties can be modulated by their monomer composition, they are produced by biological processes and therefore completely biodegradable without pollution emissions, and their degradation monomers are less acidic than those of petroleum-based bioplastics, which makes them more biocompatible [23,24]. Previous studies incorporated silver nanoparticles into poly(3-hydroxybutyrate) (PHB), using *C. necator* metabolism; however, this polymer possesses some drawbacks for manufacturing due to its brittleness and crystallinity, given its chemical composition as an aliphatic polyester with C3 lateral chains (short-chain length PHA). From the same family, poly(3-hydroxyoctanoate) (PHO), a PHA with C8 lateral chains (medium chain length), has been recognized to show better mechanical properties for manufacturing, comparable to those of synthetic plastics such as polypropylene, and can be used as an elastic polymer for several applications [25,26].

In this work, PHO bioactive films were prepared with silver and copper oxide nanoparticles as antimicrobial agents against MRSA, using *Pseudomonas putida* KT2440 as a microbial factory for the synthesis of the PHO polymer and the reduction of silver nitrate to silver nanoparticles. *P. putida* is the most studied PHO producer; it has been recognized as safe (GRAS certificate), and its metabolism and genetic capabilities are well known [27,28]. The physical-chemical characterization of the generated films was undertaken to determine the effect of the particles on the polymer’s molecular weight and thermal properties. The biocompatibility of the active PHO films was assessed through cell viability and genotoxicity in human fibroblast cells (HFF-1), including tests to evaluate the generation of DNA strand breaks (SB), oxidative DNA damage, hemolysis, and platelet aggregation in human blood samples.

## 2. Materials and Methods

### 2.1. Materials and Bacterial Strains

The MRSA strain used was obtained from the Microbial Research Center (CIMIC, Bogota, Colombia). *P. putida* was provided by the Research Group Biotechnology of Polymers from the Biological Research Center (CIB, Madrid, Spain). Copper oxide nanoparticles (CuONPs) powder was purchased from Hefei Quantum Quelle Nano Science & Technology Co., Ltd. (Hefei, China). Tris [tris(hydroxymethyl)aminomethane], Chloroform stabilized with ethanol (technical grade), DMSO [dimethyl sulfoxide], methanol (For analysis, ACS, ISO), NaOH, 1% Triton X-100, 2.5 M NaCl and EDTA [ethylenediaminetetraacetic acid] were used for all the experiments.

### 2.2. Synthesis of PHA Using Pseudomonas putida KT2440

Single colonies of *P. putida* were transferred from LB agar plates (Luria Bertani, Neogen, Lansing, MI, USA) to Erlenmeyer flasks of 2 L containing 800 mL of LB broth as a starting culture. The cultures were incubated at 30 °C and 200 rpm for 24 h. For the synthesis of intracellular PA granules, cells from LB broth were transferred to 0.1N M63 medium, which is a nitrogen-limited minimal medium consisting of KH_2_PO_4_ (13.6 g/L), (NH4)_2_SO_4_ (0.2 g/L), and FeSO_4_·7H_2_O (0.5 mg/L), 1 mM MgSO_4_, and 1 mL/L of a solution of trace elements (composition 1000×, 2.78 g of FeSO_4_·7H_2_O g/L, 1.98 g of MnCl_2_·4H_2_O g/L, 2.81 g of CoSO_4_·7H_2_O g/L, 1.47 g of CaCl_2_·2H_2_O g/L, 0.17 g of CuCl_2_·2H_2_O g/L, 0.29 g of ZnSO_4_·7H_2_O g/L). The pH of the solutions was adjusted to pH 7.0 with KOH. Five different protocols for PHA synthesis were tested (Figure S1, Supplementary Materials), including the ones described in [29,30,31]. The highest PHA yields were obtained with protocol N°5, when LB starting cultures were incubated for 48 h and transferred to 0.1N M63, where the carbon source was batch-fed every two hours using 1.63 mL/L of sodium octanoate 1 M. After 8 h at 30 °C and 200 rpm, 12.5 mL/L sodium octanoate (1 M) was added to obtain a final concentration of octanoate of 20 mM. The total PHA production time was 24 h.

The extraction of the biopolymer was done with organic solvents using a slightly modified method described by Castro-Mayorga et al. [16]. First, the culture media was centrifugated for 15 min at 700× *g* (Sorvall™ ST 16, Fiberlite™ F15-6x100y, Thermo Fisher, Scientific, Waltham, MA, USA) and pellets, frozen at −80 °C, at least for 24 h, freeze-dried. Then, the lyophilized biomass was transferred to chloroform and stored for 20 h at 50 °C in a closed container under magnetic stirring. After that, distilled water was added to the previous solution, and the mixture was centrifuged for 5 min at 290× *g* (Sorvall™ ST 16, Fiberlite™ F15-6x100y, ThermoFisher, Scientific, Waltham, MA, USA) to recover the polymer from the organic phase. The total polymer content (% *w*/*w*) was calculated with Equation (1) below

(1) $$\mathrm{Total}\mathrm{polymer}\mathrm{content}(\%)=\frac{extracted\;polymer\;weight}{lyophilized\;biomass\;weight}\times 100$$

The purity of the extracted polymer and its monomer composition were determined by gas chromatography-mass spectrometry (GC-MS), for which an acid methanolysis was done [16]. This methodology consists of suspending approximately 6 mg of PHA in 2 mL of 15% (*v*/*v*) H_2_SO_4_ acidified methanol containing 0.5 mg/mL of 3-methyl benzoate (internal standard) and 2 mL of chloroform. The mixture is then transferred to a tube and incubated for 4 h at 100 °C. After incubation, 1 mL of distilled water was added to induce phase separation. The methyl ester of monomers suspended in the organic phase was analyzed by GC-MS with an Agilent (Waldbronn, Germany) Series 7890A coupled with a 5975C MS detector (EI, 70 eV) and a split-splitless injector. 1 µL of the sample was injected (split ratio: 1:50) with helium as a carrier gas, maintaining the temperature at 80 °C for 2 min. During the experiment, the temperature was increased to 250 °C at a rate of 15 °C min^−1^ for efficient separation of the peaks. The injector and detector temperatures were set at 280 °C and 250 °C, respectively. Pure PHO polymer was used as a standard.

### 2.3. Biosynthesis of AgNPs (Bio-AgNPs)

The synthesis of bio-AgNPs by PHO-accumulating *P. putida* KT 2440 was performed following the procedure described in the Spanish PatentES2647324B1 [29]. Pre-cultures of *P. putida* KT2440 were prepared in LB and transferred to 0.1N M63 as described above. Following the protocol developed by Castro-Mayorga et al. [16], the cultures were treated with different concentrations of 2 and 3.5 mM to identify the best concentration for the antimicrobial activity. After 24 h of growth, the silver nitrate was added to the medium, and the reaction was incubated for 16 h at 30 °C and 200 rpm in darkness [16].

### 2.4. Preparation of Bioactive Films Containing CuONPs or Bio-AgNPs

To fabricate casted films, solutions of the polymer synthesized with AgNPs nanoparticles were prepared by dissolving 9.5% (*w*/*w*) in chloroform and stirring until complete homogenization at 50 °C. For copper-based films, CuONPs were added to the polymer solution to reach a concentration of 0.07% (*w*/*w*). The solutions were placed in Petri dishes and air-dried for 48 h to remove any remaining solvent. PHO films without metal nanoparticles were used as a control.

### 2.5. Antimicrobial Activity of Bioactive Films Containing CuONPs or Bio-AgNPs

The antimicrobial activity of the active films was tested according to ISO 22196:2011/JIS Z 2801 with some modifications [30]. Briefly, a MRSA suspension of 5 × 10^5^ CFU/mL was placed onto film samples and covered by an inert piece of low-density polyethylene (LDPE). After incubation for 24 h at room temperature and 95% RH, bacteria were recovered, and the number of viable cells was determined by plate counting. As a negative control, films without nanoparticles were used. Three specimens of each sample were tested.

### 2.6. Characterization of Bioactive Films Containing CuONPs or Bio-AgNPs

Thermogravimetric Analysis (TGA) were performed under nitrogen atmosphere in a TGA 550 thermobalance (TA Instruments, Toronto, ON, Canada). Samples were heated from room temperature up to 600 °C at a rate of 10 °C min^−1^ under nitrogen. Derivative TGA curves (DTG) express the weight loss rate as a function of temperature and the temperature of the maximum rate of degradation (Td). Fourier-transform infrared spectroscopy (FTIR) characterization was performed on samples of the PHO films. The FTIR spectra were obtained on a Nicolet 5700 FTIR spectrometer (Thermofisher, Waltham, MA, USA) in the region from 1800 to 1000 cm^−1^. Differential Scanning Calorimetry (DSC) analysis was conducted with a DSC 8000 (Perkin-Elmer Inc., Waltham, MA, USA) equipped with an intracooler system. The operation was performed on approximately 3 mg of each sample at a heating rate of 10 °C min^−1^, from −70 °C to 200 °C. The DSC equipment was calibrated using indium as a standard, and the slope of the thermograms was corrected by subtracting similar scans of an empty pan. All tests were done in triplicate. For molecular weight analysis (number average molecular weight and weight average molecular weight, Mn and Mw, respectively), a multidetector gel permeation chromatography system consisting of a precolumn, two 300 mm × 0.8 mm M columns (PSS, Mainz, Germany), an isocratic pump 2080, an automatic injector AS 2050 (both Jasco, Tokyo, Japan), a RI detector Shodex RI-101 (Showa Denko, München, Germany), and a dual detector T60A (Viscotek Corporation, Houston, TX, USA), was used. Briefly, chloroform was used as an eluent with a flow rate of 1 mL/min at 35 °C and 0.2% toluene as the internal standard. Polystyrene samples were used for universal calibration. All samples were prepared at a concentration of 2 mg/mL.

### 2.7. Preparation of Soluble Extracts for Biocompatibility Assays

The bioactive films were surface-sterilized with 70% ethanol and exposed to UV light overnight. Then films were washed three times with sterile Phosphate Buffered Saline (PBS), following the ISO109935 [31]. A 1 × 1 cm (0.219 ± 0.009 g) piece of the PHO, PHO-CuONPs, and PHO-Bio-AgNPs films were placed in 1 mL of Dulbecco’s Modified Eagle Medium (DMEM, Sigma, St. Louis, MO, USA) without serum and 1 mL of PBS 1X for the cytotoxicity and the genotoxicity evaluations, respectively. In both cases, films were incubated in a humidified 5% CO_2_ atmosphere at 37 °C for 48 h. The negative control consisted of one tube of DMEM or PBS 1X without polymer films.

### 2.8. Biocompatibility Assays

The HFF-1 cells (ATCC^®^ SCRC1041™, Manassas, VA, USA) were cultured in DMEM and supplemented with 1% penicillin/streptomycin (GIBCO, Waltham, MA, USA) and 15% heat-inactivated fetal bovine serum (GIBCO, Waltham, MA, USA). Cells were incubated in a humidified 5% CO_2_ atmosphere at 37 °C for 24 h (a complete cell cycle) before treatment. A colorimetric method with 3-[4,5-dimethylthiazol-2-yl]-2,5-diphenyltetrazolium bromide (MTT, Sigma, St. Louis, MO, USA) was used to measure viability. The test was performed in HFF-1 cells exposed to extracts obtained from the PHO, PHO-CuONPs, and PHO-Bio-AgNPs films. In 96-well flat-bottomed plates, 1 × 10^5^ cells per well were grown with three replicate wells for each treatment. The negative control consisted of 100 µL of DMEM without serum, and the positive control was medium with Triton X-100 at 1%. The plates were incubated in a humidified 5% CO_2_ atmosphere at 37 °C for 24 h. After this period, 10 µL of MTT (5 mg/mL) were added to each well, and cells were incubated for two additional hours. Then, 100 µL of DMSO were added. Formazan crystals were dissolved in agitation, and after 5 min, the plate was analyzed in a BioRad microplate reader (BioRad, Hercules, CA, USA) at 595 nm and a reference wavelength of 655 nm. The results were expressed as the percentage of living cells calculated from MTT reduction, assuming the absorbance of control cells as 100% [31].

Oxidative DNA damage was measured by the formamidopyrimidine DNA glycosylase (Fpg)-modified Comet assay. The HFF-1 cells were seeded at 3 × 10^5^ cells per mL in 96-well flat-bottomed plates in duplicates and incubated for 24 h. Subsequently, cells were treated with the film extracts for 3 h. A negative control of PBS 1X and a positive control of H_2_O_2_ (25 μM in PBS 1X, 5 min treatment at 4 °C) were included. Cells were treated for 3 h at 37 °C in a 5% CO_2_ atmosphere. After treatment, cells were washed, trypsinized, and re-suspended in a supplemented medium. The Trypan Blue dye exclusion assay for acute cytotoxicity evaluation was performed before the genotoxicity assay. The Comet assay was performed according to the procedure of Collins et al. (2002) [32], with some modifications. Two slides per well were prepared and placed overnight at 4 °C in freshly prepared lysing solutions (2.5 M NaCl, 100 mM EDTA, 10 mM Tris, 10% DMSO, 1% Triton X-100, pH 10). After lysis, slides were washed with enzyme reaction buffer (40 mM HEPES, 0.1 M KCl, 0.5 mM EDTA, 0.2 mg/mL bovine serum albumin (BSA), pH = 8.0) and incubated for 30 min at 37 °C in either enzyme buffer or buffer with Fpg enzyme. All slides were placed in an alkaline buffer (300 mM NaOH, 1 mM EDTA, pH > 13, 4 °C) in an electrophoresis unit for 25 min. Electrophoresis was conducted at 25 V and 290 mA for 35 min at 4 °C. Following electrophoresis, the slides were washed with neutralizing buffer (0.4 M Tris, pH 7.5), dehydrated with ethanol, and dried at room temperature. Gel Green 3X (Biotium, Fremont, CA, USA) was used to stain slides, and they were examined at 100× magnification with a Zeiss fluorescence microscope with an excitation filter of 495 nm and an emission filter of 517 nm. A total of 100 cells were examined per enzyme and active film extract treatment (50 cells per slide). DNA damage was measured according to the percentage of DNA in the tail (tail DNA) using the CometScore software (TriTek Corp., Sumerduck, VA, USA). The no enzyme treatment slides provide an estimate of the background SB. The enzyme-treated slides show both SB and oxidized bases (SB+OX). Assuming a linear dose response, the subtraction of SB from SB+OX gives a measure of Fpg-sensitive sites in the DNA that is representative of the extent of base oxidation.

### 2.9. Hemocompatibility

Human blood hemolysis tests were performed to evaluate red blood cells’ responses to contact with the films [33,34]. An anonymous donor blood sample of 25 mL was collected in EDTA-coated K_2_ tubes after signing and informed consent (Ethics Committee at the University of the Andes, minute number 928-2018). Tubes were centrifuged at 12× *g* (Sorvall™ ST 16, Fiberlite™ F15-6 × 100y, Thermo Fisher, Scientific, Waltham, MA, USA) for 5 min. The plasma was discarded, and the cells were washed with 0.9% (*w*/*v*) saline solution three times for a final PBS 1X wash. Later, in a 96-well plate, erythrocyte dilutions, until reaching a concentration of 0.4% (*v*/*v*) per well, were made in PBS 1X. 5 mm^2^ pieces of the neat PHO, PHO-CuONPs, and PHO-Bio-AgNPs films were placed in the wells to get in contact with the erythrocytes. A negative control of red cells with PBS 1X, and a positive control of red cells with Triton X-100 were included. The seeded plate was incubated for 1 h at 37 °C with 5% CO_2_. After incubation, each well was centrifuged at 105× *g* (Sorvall™ ST 16, Fiberlite™ F15-6 × 100y, Thermo Fisher, Scientific, Waltham, MA, USA) for 5 min. A sample of 75 µL of the supernatant was measured at 450 nm. The percentage of hemolysis for each treatment was calculated with the following equation

(2) $$H\left(\%\right)=\frac{Abs\left(s\right)-Abs\left(nc\right)}{Abs\left(ps\right)-Abs\left(nc\right)}\times 100$$

where *Abs (s)*, *Abs (nc)*, and *Abs (ps)* correspond to the absorbance at 450 nm of the test sample, the negative control, and the positive control, respectively. The average absorbance of triplicate values per treatment was analyzed.

Platelet aggregation trials were performed to evaluate blood plasma coagulation on contact with the bioactive films [33,35] in an anonymous donor blood sample. The blood sample was centrifuged at 12× *g* (Sorvall™ ST 16, Fiberlite™ F15-6 × 100y, Thermo Fisher, Scientific, Waltham, MA, USA) at room temperature for 15 min. An aliquot of 100 µL was taken for each treatment after platelet-rich plasma separation. Pieces of 5 mm^2^ of each film were placed in a 96-well plate with plasma in triplicates and incubated for 3 min at 37 °C. Oxidized films with immobilized epinephrine were used as the positive control, and the films with immobilized heparin were used as the negative control. Absorbance was read at 620 nm after the incubation period and the removal of the films. The final values were reported in terms of transmittance.

### 2.10. Statistical Analysis

The statistical analyses were performed utilizing Prism 8 (GraphPad Software, San Diego, CA, USA) and R Studio 1.2.1335 (R Studio Inc., Boston, MA, USA) for the analysis of variance (ANOVA). Homogeneous sample groups were obtained by using Tukey’s honestly significant difference with a 95% significance level.

## 3. Results and Discussion

### 3.1. Synthesis of PHA Using P. putida KT2440

A total polymer content of 38.92 ± 4.02% was obtained after extraction. The purity of the sample calculated by GC-MS was 99.6 ± 6.0 (*w*/*w*), and monomers from C8 to C10 were detected. The PHA monomeric composition was determined to be 90.3 ± 0.4% of C8 poly(3-hydroxy-octanoate) (PHO), 7.4 ± 0.4% of C6 poly(3-hydroxy-hexanoate) (PHHx), and 2.4 ± 0.1% of C10 poly(3-hydroxy-decanoate) (PHD). For practical purposes, we used the term PHO to refer to the copolymer of PHO (90.3%)-*co*-PHHx(7.4%)-*co*-PHD (2.4%). The molar composition of PHO is comparable to that of PHAs synthesized by other *Pseudomonas* species when grown on octanoate [25,36].

The polymer showed a flexible nature along with high transparency (Figure S2, Supplementary Materials), opposite to the high degree of crystallinity and rigidity characterized by PHB. The tendency of the polymers to crystallize depends on the sequence structure. PHA containing repeat units with longer side chains, such as PHO and copolymers, usually have lower crystallinities and can even be fully amorphous [37]. Moreover, PHO has been stated as a promising material because it is biocompatible, non-toxic, thermoplastic, and/or elastomeric, which are appealing properties for consumer and specialized applications [17,38].

### 3.2. Biosynthesis of AgNPs (Bio-AgNPs)

**Figure 1:**
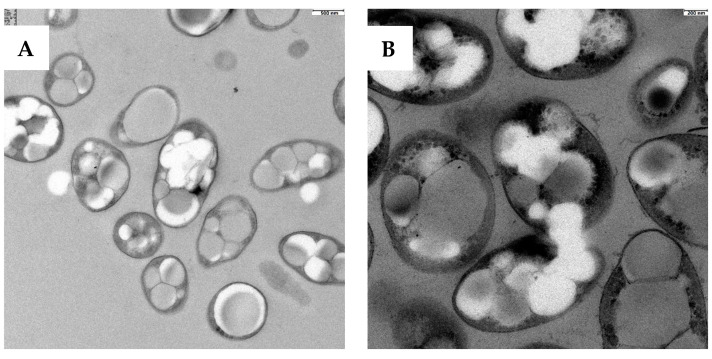
STEM micrographs of *P. putida* KT2440 cells. (**A**): untreated cells; (**B**) cells treated with 3.5 mM AgNO_3_.

Figure 1 shows a STEM image of a culture of *P*. *putida* KT2440 cells in a PHA accumulation phase, without (Figure 1A) and with the addition of AgNO_3_ (Figure 1B). As can be seen from the micrographs, electron-dense nanoparticles of spherical shape appeared inside the bacterial cytoplasm, which confirms the capacity of this strain to reduce the silver salt and to biologically synthesize silver nanoparticles (Bio-AgNPs). The fact that these NPs are located mostly inside the cytoplasm corroborates our previous observations on the reductive capacity of bacterial biomass for reduction of AgNO_3_ in PHB-accumulating bacteria such as *Cupriavidus necator* [16], abolishing the use of additional reducing agents. It is important to note that the simultaneous synthesis of intracellular polymers and nanoparticles is rather unexplored, making it difficult to compare the efficiency of nanoparticle biosynthesis and incorporation into the polymer matrix with other authors.

### 3.3. Antimicrobial Activity

**Figure 2:**
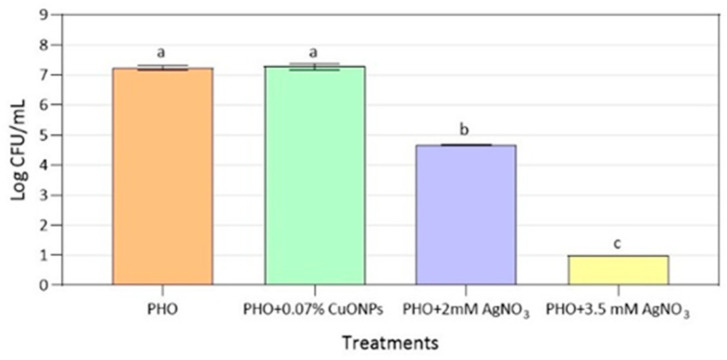
Antibacterial activity against MRSA of PHO films with different concentrations of metal nanoparticles. The detection limit was 10 CFU/mL. Significant differences (*p* < 0.05) are symbolized with different letters (a–c).

After 24 h of exposure, MRS cells showed no significant reduction in their growth on PHO films containing 0.07% (*w*/*w*) of CuONPs when compared to cell growth on PHO films without antimicrobial nanoparticles. A small reduction of 2.0 log CFU/mL was achieved with the polymer PHO + 2 mM AgNO_3_, while total inhibition of the pathogen was attained when the cells were exposed to the PHO + 3.5 mM AgNO_3_ films_,_ which were determined to be the most efficient antimicrobial material against MRS (Figure 2). Although we observed almost complete inhibition of MRSA growth on PCL films supplemented with 0.07% (*w*/*w*) CuONPs [30], the antimicrobial activity of CuONPs was reduced when mixed with PHO polymer. This could be explained by the differences in the properties of the films, and PCL hydrophilicity could provide a better environment for CuONPs’ activity [39]. On the contrary, PHO has a much more hydrophobic nature, thus decreasing the release of the metallic ions and therefore the concentration of the antimicrobial agent to which MRSA cells are exposed [40].

### 3.4. Characterization of Bioactive Films

**Figure 3:**
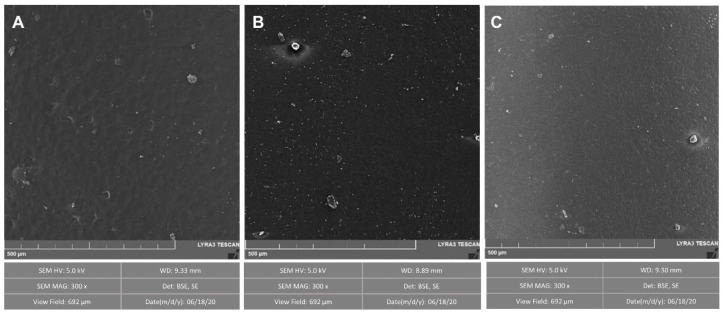
SEM micrographs of PHO films functionalized with metal nanoparticles. (**A**): PHO; (**B**): PHO + 3.5 mM AgNO_3_; (**C**): PHO + 0.07% CuONPs.

The PHO and bioactive PHO films were characterized microscopically and via thermomechanical tests to study the effect of the nanoparticles on the properties of the films. The surface of PHO films has a smooth appearance (Figure 3). Surface measurements by SEM and EDS analysis showed no signal of the nanoparticles in the outer layer, most likely due to the low concentration of copper and silver on the film surface. Although the exact concentration of silver embedded in PHO biosynthesized films is difficult to quantify, it could be estimated that the maximum silver concentration incorporated in the PHO is around 14% (*w*/*w*), based on the total polymer extracted from the *P. putida* culture (0.63 g/L) and the initial volume and concentration of the AgNO_3_ solution added during Bio-AgNPs synthesis (267 mL of 3.5 mM AgNO_3_). However, as described in our previous work, where the same AgNO_3_ concentration was used for the biosynthesis of PHB-AgNPs by *C. necator* [16], the polymer purification process after extraction drastically reduces the NPs content up to 0.015% (*w*/*w*). Thus, a more rigorous purification and characterization effort should be made in the future to quantify the amount of total silver in the active films and maybe differentiate between AgNPs and Ag+ by using methods such as an inductively coupled plasma mass spectrometer (HPLC-ICP-MS) or high-resolution continuum source graphite furnace atomic absorption spectrometry (HRCS SS GFAAS) [41,42,43].

The weight-average molecular weight (Mw) and number-average molecular weight (Mn) of the samples measured by GPC (Table 1) were approximately 60,000 and 110,000 g/mol, respectively, and had a polydispersity index (Mw/Mn) of 1.8, an acceptable PDI for biologically synthesized polymers [44]. The high molecular weight characteristic of PHA polymers was preserved during the biosynthesis of Bio-AgNPs, placing *P. putida* as a good biorefinery for PHO-nanoparticle composites.

The characterization of the films by FTIR showed no significant differences between the bare PHO and the PHO-Bio-AgNPs films. The spectra (Figure S3, Supplementary Materials) show distinct bands corresponding to the stretching bands of the ester carbonyl group C=O at 1730–1740 cm^−1^ and the component of the stretching of CH2 and CH3, expected for PHO [45,46,47,48,49]. In the PHO + 0.07% CuONPs FTIR spectrum, additional signals are seen in the amide band at 1650 cm^−1^, as well as the CN stretching and NH bending bands at 1280 cm^−1^, and the stretching band of amine groups between 3300–3500 cm^−1^. These coincide with our previous observations of the presence of proteins in the polymeric film remaining during the extraction process [18]. It should be noted that although no peaks for the nanoparticles were observed by FTIR, probably due to the low silver content, their presence was demonstrated by the antimicrobial activity tests.

**Figure 4:**
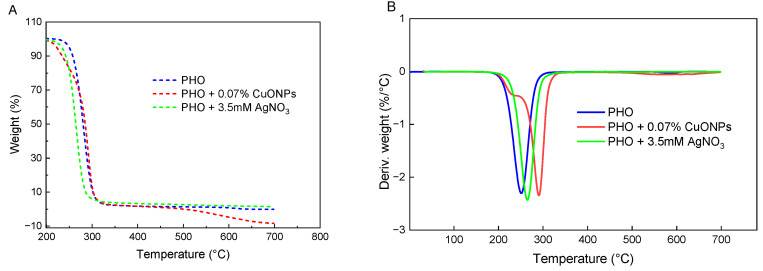
Thermal stability and degradation of active PHO films with a heating rate of 10 °C min^−1^ (**A**): weight loss (%), and (**B**): the first derivative of the weight loss curve (%/ °C).

Figure 4 shows the results from the TGA analyses and the peak calculation using the 1st derivative of the weight loss curve, characterized by a peak near 250 °C in the neat PHO sample with the greatest rate of change on the weight loss at 290 °C, which denotes the temperature at which the weight loss of PHO begins [45,50]. In the films containing Bio-AgNPs, the peak is shifted to lower values, with a maximum at 270 °C maintaining a similar profile as PHO alone, which can be interpreted as a decrease in the sample stability [51]. This could be a result of traces of silver nitrate oxide residues from the silver precursor used for in-situ synthesis. [18]. On the contrary, the addition of CuONPs to PHO films slightly enhances their thermal stability, with the degradation peak shifting to 300 °C. In addition, a weak shoulder is observed at 230–250 °C (probably caused by the presence of volatile components). This agrees with published data where the incorporation of CuONPs into polymers was seen to act as a stabilizing agent [52,53,54].

**Figure 5:**
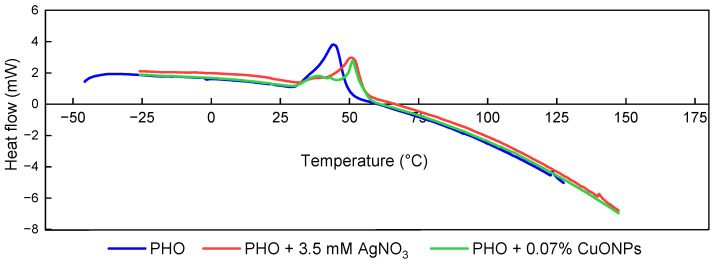
DSC thermograms of the first heating run of PHO films with and without NPs.

DSC analysis showed that the melting endotherm point was shifted to a higher temperature in the samples with metallic nanoparticles when compared to PHO, 51 °C for both PHO-CuONPs and PHO-Bio-AgNPs films (Figure 5 and Table 2). This melting temperature coincides with the 51 °C reported by Marois et al. [55] as the mean melting temperature for a sample of PHO that has not been placed under any kind of pre-degradation treatment. The result obtained also agrees with the one reported by Sofińska et al. [45], who established a temperature of 52.8 °C as the melting peak for a sample of PHO that was synthesized from the same strain as the one used in this research. Another study conducted by L. John R. Foster et al. [56] stated a temperature of around 55 °C as the mean melting temperature for a sample of PHO synthesized by using *Pseudomonas oleovorans*.

Table 2 presents the values of mean enthalpy obtained for the polymeric samples, as well as the initial temperature (Ti), the maximum temperature (Tm), and the final temperature (Tf) of melting for each material. It is relevant to notice that PHO is considered an amorphous polymer since a value for the theoretical fusion enthalpy of 100% crystalline PHO has not been reported yet [50,55,56,57]. No studies related to the evaluation of the thermal properties of PHO with metal nanoparticles were found; however, results published by Castro et al. [16,51], in which ZnO and AgNPs were immobilized onto different PHAs, provide evidence that the addition of metal nanoparticles decreases the crystallinity levels of the analyzed samples by hampering the recrystallization during heating as an antinucleating effect.

### 3.5. Biocompatibility Assays

**Figure 6:**
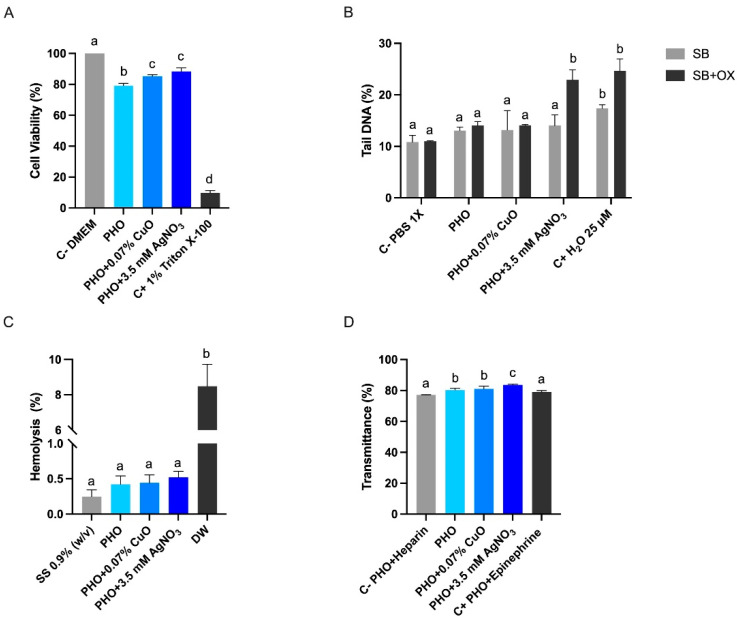
Biocompatibility of PHO active films. (**A**) viability test on HFF-1 cells after 24 h of exposure; (**B**) genotoxicity assay on HFF-1 cells after 3 h of exposure; (**C**) hemolysis caused by neat PHO films and bioactive PHO films; and (**D**) platelet aggregation of human plasma on active PHO and neat PHO films after 3 min of exposure. Mean values with different letters represent significant differences (*p* < 0.05). C− (negative control), C+ (positive control), SS (saline solution), and DW (distilled water).

To elucidate if the mechanisms by which metal nanoparticles exert an inhibitory effect in MRSA are related to mitochondrial damage, MTT assays were conducted, correlating the presence of viable cells with the appearance of a blue color, a product of the transformation of the tetrazolium ring [58]. After 24 h of exposure to the extracts from the bioactive films, HFF-1 cells exhibited cell viability above 79% for all treatments, normalized to the negative control DMEM without NPs (Figure 6A). Similarly, all treatments showed a percentage of viable cells above 80% after a 3-h treatment on the acute cytotoxicity test.

The Fpg-modified comet assay was used to measure the induction of DNA damage according to the tail DNA. Induction of SB was not observed for the bioactive film exposure, and HFF-1 cells showed only significant differences for the positive control (Figure 6B). Regarding oxidative DNA damage, the PHO + 3.5 mM exposure increases the oxidized DNA damage in HFF-1 cells (Figure 6B). It has been demonstrated that AgNPs and silver ions generate free radicals or may disrupt antioxidant mechanisms by binding enzymes and increasing reactive oxygen species (ROS) concentrations in cells [52]. The Fpg enzyme is involved in DNA repair and recognizes 8-oxo-7,8-dihydroguanine (8oxoG), creating an apurinic site, which will result in a DNA break measured by the Comet assay, and in turn, the 8-oxoG is a biomarker of ROS-induced DNA damage [53]. In human cells, the 8-hydroxy-2′-deoxyguanosine (8-oxo-dG), a deoxyriboside form of 8-oxoG, level increases after AgNPs exposure at doses above 25 µg/mL [54]. Thus, the observed antibacterial activity of AgNPs may be explained by the increased intracellular effect of ROS and the corresponding lipid peroxidation when membrane binding subsequently causes cell death [52].

Previous studies carried out to evaluate the biocompatibility of PHO have reported positive results related to the use of this material in the biomedical area. For example, Beaulieu et al. [59] evaluated the biocompatibility of PHO when impregnated in artery prostheses in rats, reporting good behavior in terms of enzymatic activity and tissue reaction, as well as a low rate of degradation after 6 months of study. Similarly, Nurlu et al. [38] implemented this biopolymer for the repair of gaps in nerves, indicating that after 60 days of study there was regeneration of the treated tissue with minimal degradation of the material and with a minimal inflammatory response.

### 3.6. Hemocompatibility Assays

As mentioned above, when working on the development of new materials for the biomedical area, these should be biocompatible, present the minimum risk of an immune response and cellular cytotoxicity, and recover adequately from injuries [60,61]. In this sense, PHAs have been widely studied for their use for therapeutic purposes due to characteristics such as their high biocompatibility, controlled biodegradability, and the capacity to adapt their mechanical and thermal properties [62]. They are used for the development of cardiac valves, in the repair of bone, nerve, and cartilage tissue, and for the development of wound dressings [63].

Therefore, to evaluate the reaction of blood samples and their cells when contacting the active PHO films, in vitro studies of hemolysis and platelet aggregation were carried out. Figure 6C presents the results obtained for the hemolysis tests, indicating that all PHO samples, both control films and those functionalized with nanoparticles, were hemocompatible since they presented similar values to those obtained with solutions used in cell culture work, such as saline solution. In addition, all samples had a red blood cell breakage rate of less than 5%, which showed that they meet the standards established by ISO 10993-4:2002 for materials used in the biomedical area [31].

Concerning platelet aggregation, both the control PHO film and the active PHO film with nanoparticles showed similar transmittance values to those obtained with the films used as a positive control, which had epinephrine immobilized on the surface of the material (Figure 6D). This is a positive result since, according to Padalhin et al. [35], it is desired that the materials used as wound dressing allow platelet aggregation since this will support a better wound recovery due to the release of growth factors and repair signals in the affected area. It should be emphasized that a stable control comprised of PHO film with epinephrine or heparin immobilized on the surface was not obtained for the assays, making it necessary to improve the procedure for further tests.

Regarding the biocompatibility of PHO for its use in the biomedical area, Witko et al. [64] studied the physiological and morphological impact on fibroblasts when using PHO films as substrate, describing cell viability above 90% when chloroform was used as the extraction solvent and after 24 h of the drying process. This differs from the 80% cell viability obtained in our study due to shorter drying times after the solvent extraction, which could lead to chloroform residue. Similarly, fibroblasts exhibited average cell viability higher than 80% when exposed to PCL films with and without CuONPs [30].

## 4. Conclusions

Antibiotic resistance is a threat to the well-being of the human population. Numerous efforts are being conducted in the field to find new antibiotics, vaccines, and monitoring strategies to prevent and treat infections with multiresistant microbes. In this study, we demonstrated that *P. putida* KT2440 can reduce AgNO_3_ to Ag nanoparticles, which were then incorporated into the in-situ synthesized aliphatic polyester PHO, an elastic, bio-based, and biocompatible polymer from the PHA family. PHO-Bio-AgNPs films were shown as promising candidates for the design and manufacturing of antimicrobial wound dressings to treat recurrent skin and soft tissue infections, as evidenced by their antibacterial capacity against MRSA and the retention of the molecular properties of the polymers in the presence of inorganic nanoparticles. Furthermore, PHO biodegradation products have been shown to be non-toxic; thus, these films could be expanded to long-term applications where material degradation is desired. The potential of these films to treat other microbial infections needs to be further studied, which could aid in their exploitation as an alternative wound dressing.

## 5. Patents

The synthesis of Bio-AgNPs by *P. putida* KT 2440 was based on a procedure described in the Spanish Patent ES2647324B1 [29].

## Figures and Tables

**Table 1:** Gel permeation chromatography (GPC) of the neat PHO and bioactive films at 35 °C.

Samples	Mw (g/mol)	Mn (g/mol)
PHO	6.148 × 10^4^	1.308 × 10^5^
PHO + 0.07% CuONPs	5.688 × 10^4^	1.118 × 10^5^
PHO + 3.5 mM AgNO_3_	5.742 × 10^4^	1.305 × 10^5^

**Table 2:** DSC maximum of melting (Tm) and melting enthalpy (∆Hm) calculated from the first heating scan of PHO films.

Samples	Ti (°C)	Tf (°C)	Tm (°C)	∆Hm (J/g PHO)
PHO	26.2 ± 0.2	58.5 ± 0.5	43.6 ± 0.8 ^a^	26.0 ± 1.3 ^a^
PHO + 0.07% CuONPs	24.8 ± 0.6	63.2 ± 0.6	51.0 ± 0.2 ^b^	24.8 ± 0.5 ^a^
PHO + 3.5 mM AgNO_3_	30.1 ± 0.6	63.8 ± 1.1	51.9 ± 1.5 ^b^	19.9 ± 0.5 ^b^

The standard deviation was calculated from three measures. Sample mean with different superscript letters (a, b) in the same column are significantly different at *p* < 0.05.

## Data Availability

The data presented in this study are available in this article or in the supplementary material.

## Supplementary Materials

The following supporting information can be downloaded at: https://www.mdpi.com/article/10.3390/polym15040920/s1. Figure S1: Protocols tested to increase the yields of PHO biosynthesized from liquid culture of *P. putida* KT2440; Figure S2: Macroscopic aspect of PHO film obtained from *P. putida* KT2440; Figure S3: FTIR spectra of neat PHO film and active PHO films with different concentrations of metal nanoparticles.

## References

[1] Ong P.Y. (2014). Recurrent MRSA Skin Infections in Atopic Dermatitis. J. Allergy Clin. Immunol. Pract..

[2] Silva V., Peirone C., Capita R., Alonso-Calleja C., Marques-Magallanes J.A., Pires I., Maltez L., Pereira J.E., Igrejas G., Poeta P. (2021). Topical Application of Ozonated Oils for the Treatment of MRSA Skin Infection in an Animal Model of Infected Ulcer. Biology.

[3] Lee A.S., De Lencastre H., Garau J., Kluytmans J., Malhotra-Kumar S., Peschel A., Harbarth S. (2018). Methicillin-Resistant Staphylococcus Aureus. Nat. Rev. Dis. Prim..

[4] Henciya S., Vengateshwaran T.D., Gokul M.S., Dahms H.U., James R.A. (2020). Antibacterial Activity of Halophilic Bacteria Against Drug-Resistant Microbes Associated with Diabetic Foot Infections. Curr. Microbiol..

[5] Turner N.A., Sharma-Kuinkel B.K., Maskarinec S.A., Eichenberger E.M., Shah P.P., Carugati M., Holland T.L., Fowler V.G. (2019). Methicillin-Resistant Staphylococcus Aureus: An Overview of Basic and Clinical Research. Nat. Rev. Microbiol..

[6] Gianino E., Miller C., Gilmore J. (2018). Smart Wound Dressings for Diabetic Chronic Wounds. Bioengineering (Basel, Switzerland).

[7] Kwiatkowska A., Drabik M., Lipko A., Grzeczkowicz A., Stachowiak R., Marszalik A., Granicka L.H. (2022). Composite Membrane Dressings System with Metallic Nanoparticles as an Antibacterial Factor in Wound Healing. Membranes (Basel).

[8] Thomas R., Soumya K.R., Mathew J., Radhakrishnan E.K. (2015). Electrospun Polycaprolactone Membrane Incorporated with Biosynthesized Silver Nanoparticles as Effective Wound Dressing Material. Appl. Biochem. Biotechnol..

[9] Marambio-Jones C., Hoek E.M.V. (2010). A Review of the Antibacterial Effects of Silver Nanomaterials and Potential Implications for Human Health and the Environment. J. Nanoparticle Res..

[10] Yin I.X., Zhang J., Zhao I.S., Mei M.L., Li Q., Chu C.H. (2020). The Antibacterial Mechanism of Silver Nanoparticles and Its Application in Dentistry. Int. J. Nanomedicine.

[11] Candidate List of Substances of Very High Concern for Authorisation—ECHA. https://echa.europa.eu/candidate-list-table/-/dislist/details/0b0236e1807dec94.

[12] Alsammarraie F.K., Wang W., Zhou P., Mustapha A., Lin M. (2018). Green Synthesis of Silver Nanoparticles Using Turmeric Extracts and Investigation of Their Antibacterial Activities. Colloids Surf. B Biointerfaces.

[13] Philip D. (2010). Honey Mediated Green Synthesis of Silver Nanoparticles. Spectrochim. Acta Part A Mol. Biomol. Spectrosc..

[14] Umer A., Naveed S., Ramzan N., Rafique M.S., Imran M. (2014). A Green Method for the Synthesis of Copper Nanoparticles Using L-Ascorbic Acid. Rev. Mater..

[15] Kulkarni N., Muddapur U. (2014). Biosynthesis of Metal Nanoparticles: A Review. J. Nanotechnol..

[16] Castro-Mayorga J.L., Freitas F., Reis M.A.M., Prieto M.A., Lagaron J.M. (2018). Biosynthesis of Silver Nanoparticles and Polyhydroxybutyrate Nanocomposites of Interest in Antimicrobial Applications. Int. J. Biol. Macromol..

[17] Kalaoglu-Altan O.I., Baskan H., Meireman T., Basnett P., Azimi B., Fusco A., Funel N., Donnarumma G., Lazzeri A., Roy I. (2021). Silver Nanoparticle-Coated Polyhydroxyalkanoate Based Electrospun Fibers for Wound Dressing Applications. Materials (Basel).

[18] Castro-Mayorga J.L., Martínez-Abad A., Fabra M.J., Olivera C., Reis M., Lagarón J.M. (2014). Stabilization of Antimicrobial Silver Nanoparticles by a Polyhydroxyalkanoate Obtained from Mixed Bacterial Culture. Int. J. Biol. Macromol..

[19] Castro-Mayorga J.L., Fabra M.J., Cabedo L., Lagaron J.M. (2016). On the Use of the Electrospinning Coating Technique to Produce Antimicrobial Polyhydroxyalkanoate Materials Containing In Situ-Stabilized Silver Nanoparticles. Nanomaterials.

[20] Castro-Mayorga J.L., Fabra M.J., Lagaron J.M. (2016). Stabilized Nanosilver Based Antimicrobial Poly(3-Hydroxybutyrate-Co-3-Hydroxyvalerate) Nanocomposites of Interest in Active Food Packaging. Innov. Food Sci. Emerg. Technol..

[21] Chen G., Wu Q. (2005). The Application of Polyhydroxyalkanoates as Tissue Engineering Materials. Biomaterials.

[22] Chung M.G., Kim H.W., Kim B.R., Kim Y.B., Rhee Y.H. (2012). Biocompatibility and Antimicrobial Activity of Poly(3-Hydroxyoctanoate) Grafted with Vinylimidazole. Int. J. Biol. Macromol..

[23] Li W., Cicek N., Levin D.B., Liu S. (2019). Enabling Electrospinning of Medium-Chain Length Polyhydroxyalkanoates (PHAs) by Blending with Short-Chain Length PHAs. Int. J. Polym. Mater. Polym. Biomater..

[24] Ramier J., Boubaker M.B., Guerrouache M., Langlois V., Grande D., Renard E. (2014). Novel Routes to Epoxy Functionalization of PHA-Based Electrospun Scaffolds as Ways to Improve Cell Adhesion. J. Polym. Sci. Part A Polym. Chem..

[25] Huisman G.W., Wonink E., Meima R., Kazemier B., Terpstra P. (1991). Metabolism of Poly(3-Hydroxyalkanoates) (PHAs) by Pseudomonas Oleovorans. J. Biol. Chem..

[26] Rehm B.H.A. (2010). Bacterial Polymers: Biosynthesis, Modifications and Applications. Nat. Publ. Gr..

[27] Prieto A., Escapa I.F., Martínez V., Dinjaski N., Herencias C., de la Peña F., Tarazona N., Revelles O. (2016). A Holistic View of Polyhydroxyalkanoate Metabolism in Pseudomonas Putida. Environ. Microbiol..

[28] Escapa I.F., García J.L., Bühler B., Blank L.M., Prieto M.A. (2012). The Polyhydroxyalkanoate Metabolism Controls Carbon and Energy Spillage in Pseudomonas Putida. Environ. Microbiol..

[29] (2016). Procedure for obtaining antimicrobial biopolymers understanding polyhydroxialcanoates and metal nanoparticles.

[30] Balcucho J., Narváez D.M., Castro-Mayorga J.L. (2020). Antimicrobial and Biocompatible Polycaprolactone and Copper Oxide Nanoparticle Wound Dressings against Methicillin-Resistant Staphylococcus Aureus. Nanomaterials.

[31] (2006). Biological Evaluation of Medical Devices Part 4—Selection of Tests for Interactions with Blood.

[32] Collins A.R., Dusinská M. (2002). Oxidation of Cellular DNA Measured with the Comet Assay. Methods Mol. Biol..

[33] Liu H.Y., Du L., Zhao Y.T., Tian W.Q. (2015). In Vitro Hemocompatibility and Cytotoxicity Evaluation of Halloysite Nanotubes for Biomedical Application. J. Nanomater..

[34] Evans B.C., Nelson C.E., Yu S.S., Beavers K.R., Kim A.J., Li H., Nelson H.M., Giorgio T.D., Duvall C.L. (2013). Ex Vivo Red Blood Cell Hemolysis Assay for the Evaluation of PH-Responsive Endosomolytic Agents for Cytosolic Delivery of Biomacromolecular Drugs. J. Vis. Exp..

[35] Padalhin A.R., Ba Linh N.T., Ki Min Y., Lee B.T. (2014). Evaluation of the Cytocompatibility Hemocompatibility in Vivo Bone Tissue Regenerating Capability of Different PCL Blends. J. Biomater. Sci. Polym. Ed..

[36] Escapa I.F., del Cerro C., García J.L., Prieto M.A. (2013). The Role of GlpR Repressor in Pseudomonas Putida KT2440 Growth and PHA Production from Glycerol. Environ. Microbiol..

[37] Tarazona N.A., Machatschek R., Lendlein A. (2020). Unraveling the Interplay between Abiotic Hydrolytic Degradation and Crystallization of Bacterial Polyesters Comprising Short and Medium Side-Chain-Length Polyhydroxyalkanoates. Biomacromolecules.

[38] Nurlu G., Balci S., Bal E., Benli K., Hazer B., Hazer D.B., Öztürk F. (2013). In Vivo Application of Poly-3-Hydroxyoctanoate as Peripheral Nerve Graft. J. Zhejiang Univ. Sci. B.

[39] Palza H., Quijada R., Delgado K. (2015). Antimicrobial Polymer Composites with Copper Micro- and Nanoparticles: Effect of Particle Size and Polymer Matrix. J. Bioact. Compat. Polym..

[40] Yang Z., Peng H., Wang W., Liu T. (2010). Crystallization Behavior of Poly(ε-Caprolactone)/Layered Double Hydroxide Nanocomposites. J. Appl. Polym. Sci..

[41] Sötebier C.A., Weidner S.M., Jakubowski N., Panne U., Bettmer J. (2016). Separation and Quantification of Silver Nanoparticles and Silver Ions Using Reversed Phase High Performance Liquid Chromatography Coupled to Inductively Coupled Plasma Mass Spectrometry in Combination with Isotope Dilution Analysis. J. Chromatogr. A.

[42] Chao J.B., Liu J.F., Yu S.J., Di Feng Y., Tan Z.Q., Liu R., Yin Y.G. (2011). Speciation Analysis of Silver Nanoparticles and Silver Ions in Antibacterial Products and Environmental Waters via Cloud Point Extraction-Based Separation. Anal. Chem..

[43] Benton E.N., Marpu S.B., Omary M.A. (2019). Ratiometric Phosphorescent Silver Sensor: Detection and Quantification of Free Silver Ions within Silver Nanoparticles. ACS Appl. Mater. Interfaces.

[44] Singh M., Kumar P., Ray S., Kalia V.C. (2015). Challenges and Opportunities for Customizing Polyhydroxyalkanoates. Indian J. Microbiol..

[45] Sofińska K., Barbasz J., Witko T., Dryzek J., Haraźna K., Witko M., Kryściak-Czerwenka J., Guzik M. (2019). Structural, Topographical, and Mechanical Characteristics of Purified Polyhydroxyoctanoate Polymer. J. Appl. Polym. Sci..

[46] Marois Y., Zhang Z., Vert M., Beaulieu L., Lenz R.W., Guidoin R. (1999). In Vivo Biocompatibility and Degradation Studies of Polyhydroxyoctanoate in the Rat: A New Sealant for the Polyester Arterial Prosthesis. Tissue Eng..

[47] Elbahloul Y., Steinbüchel A. (2009). Large-Scale Production of Poly(3-Hydroxyoctanoic Acid) by Pseudomonas Putida GPo1 and a Simplified Downstream Process. Appl. Environ. Microbiol..

[48] Jiang X., Ramsay J.A., Ramsay B.A. (2006). Acetone Extraction of Mcl-PHA from Pseudomonas Putida KT2440. J. Microbiol. Methods.

[49] Kunasundari B., Sudesh K. (2011). Isolation and Recovery of Microbial Polyhydroxyalkanoates. Express Polym. Lett..

[50] Escapa I.F., Morales V., Martino V.P., Pollet E., Avérous L., García J.L., Prieto M.A. (2011). Disruption of β-Oxidation Pathway in Pseudomonas Putida KT2442 to Produce New Functionalized PHAs with Thioester Groups. Appl. Microbiol. Biotechnol..

[51] Castro-Mayorga J.L., Fabra M.J., Pourrahimi A.M., Olsson R.T., Lagaron J.M. (2017). The Impact of Zinc Oxide Particle Morphology as an Antimicrobial and When Incorporated in Poly(3-Hydroxybutyrate-Co-3-Hydroxyvalerate) Films for Food Packaging and Food Contact Surfaces Applications. Food Bioprod. Process..

[52] Guo Z., Liang X., Pereira T., Scaffaro R., Thomas Hahn H. (2007). CuO Nanoparticle Filled Vinyl-Ester Resin Nanocomposites: Fabrication, Characterization and Property Analysis. Compos. Sci. Technol..

[53] Shankar S., Wang L.F., Rhim J.W. (2017). Preparation and Properties of Carbohydrate-Based Composite Films Incorporated with CuO Nanoparticles. Carbohydr. Polym..

[54] Zare Y., Shabani I. (2016). Polymer/Metal Nanocomposites for Biomedical Applications. Mater. Sci. Eng. C.

[55] Marois Y., Zhang Z., Vert M., Deng X., Lenz R., Guidoin R. (2000). Mechanism and Rate of Degradation of Polyhydroxyoctanoate Films in Aqueous Media: A Long-Term in Vitro Study. J. Biomed. Mater. Res..

[56] Foster L.J.R., Russell R.A., Sanguanchaipaiwong V., Stone D.J.M., Hook J.M., Holden P.J. (2006). Biosynthesis and Characterization of Deuterated Polyhydroxyoctanoate. Biomacromolecules.

[57] Panaitescu D.M., Lupescu I., Frone A.N., Chiulan I., Nicolae C.A., Tofan V., Stefaniu A., Somoghi R., Trusca R. (2017). Medium Chain-Length Polyhydroxyalkanoate Copolymer Modified by Bacterial Cellulose for Medical Devices. Biomacromolecules.

[58] Lewinski N., Colvin V.D.R. (2008). Cytotoxicity of Nanoparticles. Small.

[59] Beaulieu L., Sc B., Lenz R.W., Guidoin R. (1999). In Vivo Biocompatibility and Degradation Studies of Polyester Arterial Prosthesis. Tissue Eng..

[60] Singh A.K., Srivastava J.K., Chandel A.K., Sharma L., Mallick N., Singh S.P. (2019). Biomedical Applications of Microbially Engineered Polyhydroxyalkanoates: An Insight into Recent Advances, Bottlenecks, and Solutions. Appl. Microbiol. Biotechnol..

[61] Bunster G.F. (2016). Polyhydroxyalkanoates: Production and Use. Encycl. Biomed. Polym. Polym. Biomater..

[62] Chen Y., Hung S.-T., Chou E., Wu H.-S. (2017). Review of Polyhydroxyalkanoates Materials and Other Biopolymers for Medical Applications. Mini. Rev. Org. Chem..

[63] Zhang J., Shishatskaya E.I., Volova T.G., da Silva L.F., Chen G.Q. (2018). Polyhydroxyalkanoates (PHA) for Therapeutic Applications. Mater. Sci. Eng. C.

[64] Witko T., Solarz D., Feliksiak K., Rajfur Z., Guzik M. (2019). Cellular Architecture and Migration Behavior of Fibroblast Cells on Polyhydroxyoctanoate (PHO): A Natural Polymer of Bacterial Origin. Biopolymers.

