# Supplementary material for: Microbially Synthesized Polymer-Metal Nanoparticles Composites as Promising Wound Dressings to Overcome Methicillin-Resistance Staphylococcus aureus Infections
Source: Polymers (Basel). 2023 Feb 12;15(4):920. doi: 10.3390/polym15040920 (PMC9960834; doi:10.3390/polym15040920)

polymers

Supplementary Material

Figure S1. Protocols tested to increase the yields of PHO biosynthesized from liquid culture of *P. putida* KT2440.

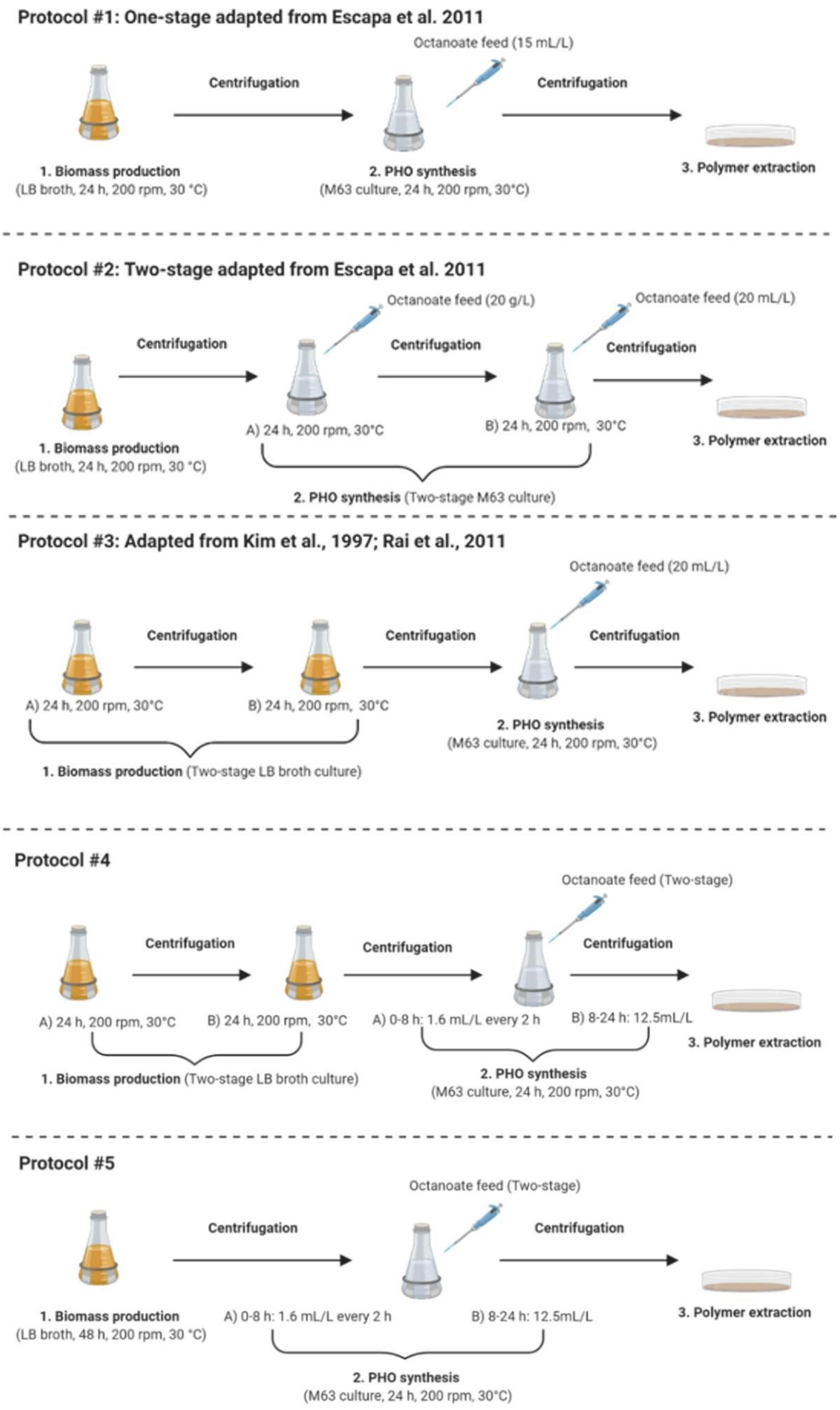

Figure S2. Macroscopic aspect of PHO film obtained from *P. putida* KT2440

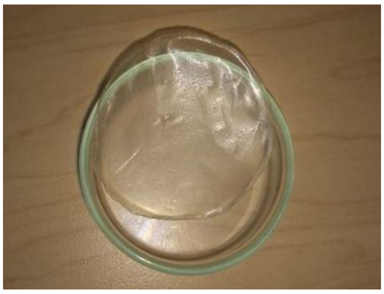

Figure S3. FTIR spectra of neat PHO film and active PHO films with different concentrations of metal nanoparticles.

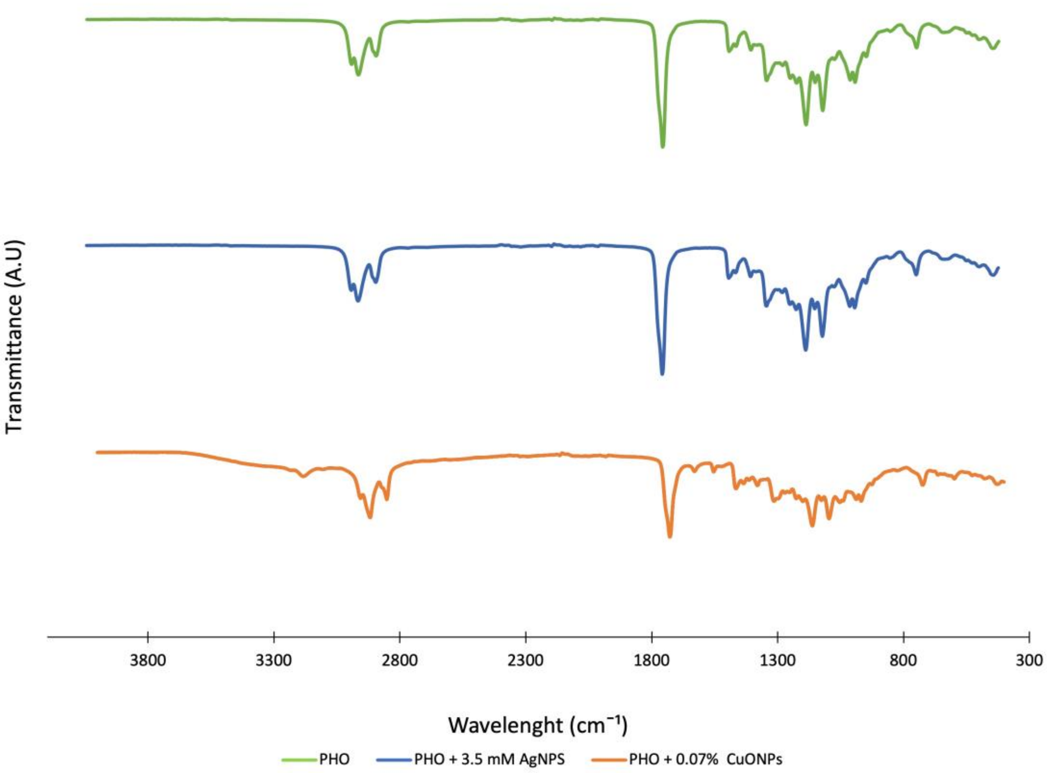

Supplement: Supplementary file 1 [file polymers-15-00920-s001.zip › polymers-2159617-supplementary.pdf]
